# The organization of spinal neurons: Insights from single cell sequencing

**DOI:** 10.1016/j.conb.2023.102762

**Published:** 2023-08-30

**Authors:** R. Brian Roome, Ariel J. Levine

**Affiliations:** Spinal Circuits and Plasticity Unit, National Institute of Neurological Disorders and Stroke, National Institutes of Health; Bethesda, MD, USA

## Abstract

To understand how the spinal cord enacts complex sensorimotor functions, researchers have studied, classified, and functionally probed it’s many neuronal populations for over a century. Recent developments in single-cell RNA-sequencing can characterize the gene expression signatures of the entire set of spinal neuron types and can simultaneously provide an unbiased view of their relationships to each other. This approach has revealed that the location of neurons predicts transcriptomic variability, as dorsal spinal neurons become highly distinct over development as ventral spinal neurons become less so. Temporal specification is also a major source of gene expression variation, subdividing many of the canonical embryonic lineage domains. Together, birthdate and cell body location are fundamental organizing features of spinal neuron diversity.

## Introduction

To understand how the spinal cord accomplishes its myriad tasks in sensation, autonomic control, and movement, we might first ask: what is the spinal cord made of? What are its parts, how should they be conceptualized, and how do they relate to each other and to the functional whole? The basic anatomical unit of the spinal cord is the segment — the neural tissue that corresponds to one vertebral bone, one set of sensory nerves relaying cues from the periphery, and one set of autonomic or motor nerves to control the organs and muscles of the body. Segments have specialized functions at different rostro-caudal levels, but they are all characterized by two shared features. First, there is a common structure. The dorsal horn includes a layered region in which each lamina is innervated by a unique complement of sensory afferents and from which ascending neurons relay sensory information to the brain [1]. The ventral horn is targeted by numerous descending motor control pathways and contains motoneurons as well as interneurons involved in the central pattern generator [2–4]. Between these two horns lies a “mid” region, dense with first order premotor neurons and thought to play a role in sensorimotor integration [5,6]. Second, each segment is populated by a common set of diverse interneuron types. (Here, “interneuron” refers to all non-motor, non-sensory neurons and includes excitatory and inhibitory, local and projection neurons.)

Spinal cord neurons have been classified using multiple schemas, including “cardinal classes” defined by the embryonic progenitor domain from which they originate [7], mature types with stereotypical circuit features such as Renshaw cells or lamina I projection neurons [8], and populations marked by particular proteins or genetic tools [1,2,6,9–12]. A complementary approach, single cell RNA-sequencing, classifies spinal neurons based on the patterns of genes that they express [13]. These signatures can be used to generate an atlas or a census of cell types in a given tissue, complete with candidate marker genes and a characterization of the molecular repertoire of each cell type. But perhaps the most important advantage of single cell experiments is that all cell types in a given tissue can be captured and profiled simultaneously, enabling their direct comparison through computational analysis. This includes dimensionality reduction to identify co-varying patterns of gene expression, clustering thousands of individual single cell transcriptomes into recurring cell types, and examining overall transcriptional similarities and differences [14,15]. What is emerging now is an updated cell type ontology for the spinal cord and beyond [16–18].

This review will focus on recent insights into spinal interneuron organization based on single cell sequencing of mammalian spinal cords. It will not delve into the diverse non-neuronal cell types, the burgeoning literature on motoneuron subtype identification [19,20], knowledge of cell type diversity along the rostro-caudal axis [21–23], or recent work in other vertebrate species [24,25]; nor will we provide detailed descriptions of individual cell types. Instead, we will emphasize the two major drivers of spinal neuron organization, as revealed by single cell experiments: the importance of *place* (such that neurons located in the postnatal dorsal horn are organized into distinct, robust types while postnatal ventral neurons are present in overlapping populations) and the importance of *time* (such that mid and ventral neurons can be sub-divided by their birthdate as well as their embryonic lineage).

### The importance of place: dorsal-ventral location defines cell type relationships

**Figure 1:**
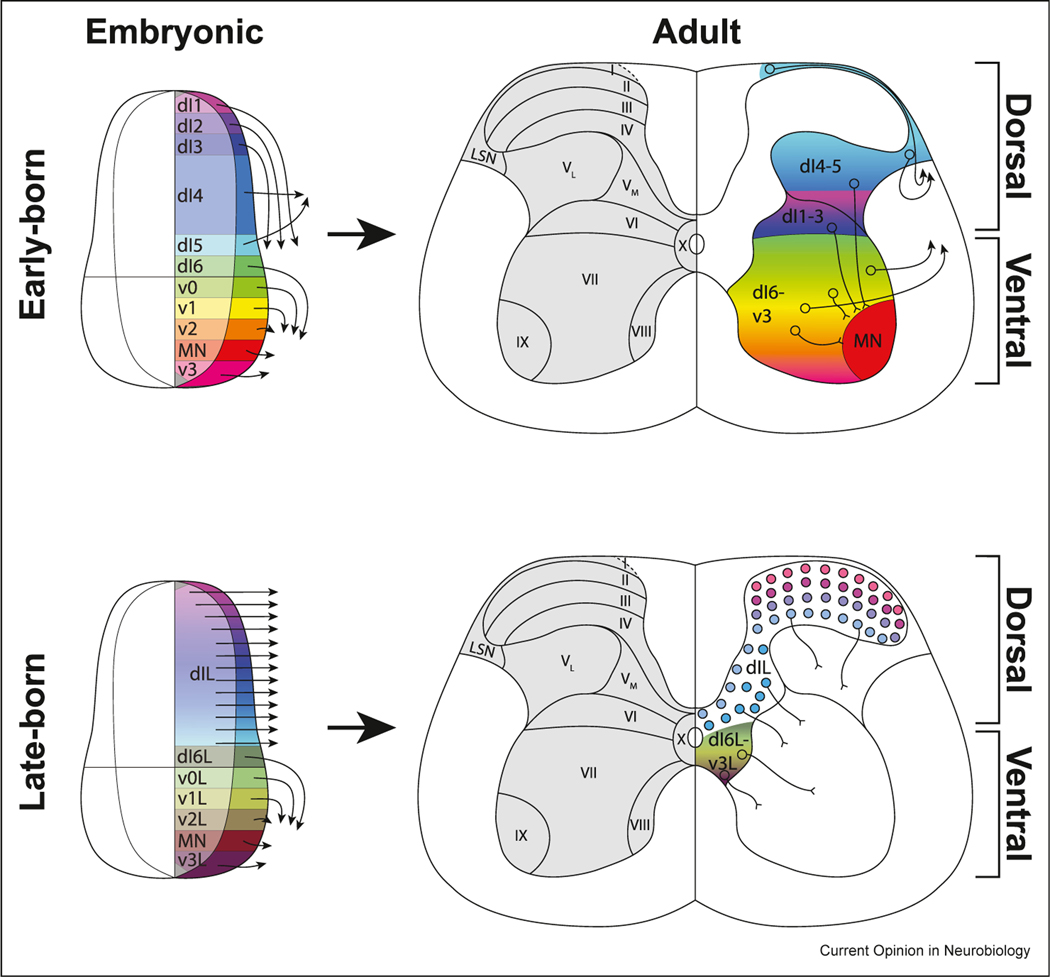
How spinal cord neuron identity varies by location and birthdate Early-born neurons of the spinal cord can be divided into cardinal groups dI1–6, v0-v3 and MN, which occupy more lateral portions of the developed spinal cord, as well as lamina I (top). During a later phase of neurogenesis, progenitors of dI1–5 transform and produce the vast types of dIL neuron, populating laminae II-IV and the medial portion of V and VI. All other progenitor domains produce analogues of their earlier versions, which settle in the medial portion of laminae VI, VII and VIII (bottom). Earlier born neurons (top) are more likely to include projection and premotor neurons, while later-born neurons (bottom) are more likely to project locally. Early-born and ventral spinal neuron types are distinct embryonically, but become transcriptionally similar in adulthood (represented by color gradients). Conversely, dorsal late-born neuron types are transcriptionally and ontogenetically similar embryonically, but become highly transcriptionally distinct in adulthood, represented by filled circles with distinct colors. Abbreviations: Roman numerals indicate the nine laminae of Rexed (I-IX), dI: dorsal interneuron, LSN: lateral spinal nucleus, MN: motor neuron, v: ventral (interneuron).

From neonatal mouse to adult human spinal cord, single cell studies have highlighted the importance of cell body settling location as the first axis of variation in clustering the diverse interneuron and projection types. The dominance of anatomical region as an organizing principle in single cell spinal cord data was first described by Sathyamurthy et al. [26]. Comparisons of the dozens of transcriptionally defined adult mouse spinal cord cell types revealed statistically robust dorsal clusters with clear markers and less robust ventral clusters with markers that overlapped multiple clusters. Rosenberg et al. developed a novel single cell sorting and molecular barcoding approach, Split-seq, and used it to profile the mouse spinal cord from two- and eleven-day old animals [27]. They also observed a wide range of dorsal clusters as well as a large, ventrally located, “unresolvable” cluster that could be not divided into clear cell types. Subsequently, a harmonized meta-analysis of six independent studies of the postnatal through adult mouse spinal cord, by Russ et al., confirmed this differential organization in a broader context [28]. A dendrogram of cell relatedness shows that the first branchpoint in the tree for all interneurons is based on dorsal versus ventral location, even before the branches that divide excitatory and inhibitory types from each other. In addition, dorsal clusters were more distant from each other in principal-component space (of gene expression variance); were more resilient than ventral clusters to repeated clustering using different parameters; and could be more reliably classified using machine learning. Osseward et al. used hierarchical clustering to probe the organization of embryonic and neonatal spinal neurons, iteratively splitting all interneurons into two groups and querying the key branch points at each split [29]. They found that the primary split divides neurons into dorsal “E” and mid-ventral “H” groups, labeled by Ebf1 and Hoxc10, respectively. Each group could then be further sub-divided by birthdate (see below), as well as neurotransmitter status and cardinal lineage class. By examining each of these features as explanators of transcriptional variance amongst spinal neurons, this study effectively ranked their contributions and confirmed spatial location as the primary factor in categorizing spinal neuron diversity. Finally, Yadav et al. extended this finding to the human spinal cord, using a range of metrics to demonstrate that dorsal cell types are discrete entities that can be easily classified and that have distinct gene expression signatures and well conserved specific homologues between species, while ventral cell types are organized more like overlapping continua with more similar gene expression across related cell types both within and between species [30] (Figure 1).

It is important to note that the differences between dorsal and ventral cell types do not simply reflect their developmental origin in the Wnt/BMP gradients of dorsal progenitors or the Shh gradients of the ventral progenitors. In fact, the neuron types derived from the most dorsal domains — the class A dI1–3 populations — are known to migrate to the mid and ventral regions of the spinal cord [31,32] (Figure 1); these populations are organized more similarly to their final neighbors in the ventral horn than their developmental “cousins” among the dorsal class B dI4–5 populations. This trend can be seen in the grouping of dI1–3 cell types in the “H” group from the Osseward study and of the putative assignment of these cell lineages into the ventral Excit-31, Excit-32, and Excit-33 postnatal-adult cell types from the Russ study [28,29].

The difficulty in resolving post-natal ventral cell types is surprising, given the rich literature of well-established genetic lineage and molecular studies that detailed dozens of distinct populations emerging from the ventral horn embryonic lineage domains. Such diversity has also been observed through single cell studies of embryonic spinal cord [33,34]. How can these two bodies of data be reconciled? One possibility is that cell type diversity is most important during development, such that factors that distinguish different cell types can be down-regulated after terminal neuronal differentiation and axon guidance. However, an analysis of fetal human spinal cord, by Zhang et al., hints that cell types derived from ventral domains and dI1–3 are already converging to a dense core in a UMAP visualization during a time when circuits are still being formed [35]. The down-regulation of cell type markers leading to an apparent blurring of cell type differences is certainly possible, but it leaves unanswered the key question of why this would impact dorsal and ventral cell types differentially. Perhaps ventrally settling neurons, which include a higher proportion of propriospinal and projection neurons, show enhanced diversity during development because each lineage domain includes locally and long-distance growing sub-types [12,29,36–39]. Perhaps dorsal neurons, which receive and send diverse peptidergic signals [40] require the sustained expression of gene expression signatures that underlie this neuro-modulatory potential.

An alternative explanation is that the different cell type relationships in the dorsal and ventral horns may be directly important in mature spinal circuits and the product of an active process. Perhaps discrete cell types in the dorsal horn support a set of specialized microcircuits with distinct functions and behavioral outputs. In contrast, ventral populations with overlapping distributions of cell properties could support robust population-scale network function, as suggested by recent work in turtle ventral central pattern generator neurons [41]. Single cell sequencing is a powerful approach to characterize and classify neuronal diversity, but now we must ask: to what extent are mature transcriptional cell types the computational units of spinal cord function and is this different in the dorsal and ventral horns?

The different organization of the dorsal and ventral regions of the spinal cord may also reflect a general principle of central nervous system organization. Indeed, a recent spatial and transcriptional atlas of millions of mouse brain cells by Yao et al. found that structures in dorsal/anterior brain regions contained very distinct cell types and ventral/posterior brain regions were made up of more closely related populations [42]. While it is difficult to compare neural features across brain regions that are completely separated anatomically, the integrated nature of dorsal and ventral cell types and circuits in the spinal cord may allow us to uncover fundamental relationships between cell type diversity and function.

### The importance of time: birthdate as an organizing principle for spinal neuron diversity

Birthdate has been shown to be a powerful organizing principle throughout the central nervous system. A classic example is the development of excitatory neurons of the cerebral cortex, which diversifies and populates progressively more superficial layers in order of their birth. Temporal diversification of neurons is directed by highly conserved transcription factor networks present in neural progenitor cells and takes place in such diverse structures as the mammalian retina, zebrafish motorneurons, and drosophila nervous system, suggesting a foundational mechanism of neuronal diversification [43–45].

Temporal diversification of spinal cord neurons has recently been shown to be a common phenomenon within all cardinal classes of spinal neurons. This was first demonstrated by Delile et al. [33], using a series of single-cell RNA-sequencing data sets from progressively older embryonic mouse spinal cords. The authors demonstrated that Onecut-positive neurons predominated in E9.5-derived data, while Zfhx-positive neurons emerged at E10.5-E11.5, and finally Nfi-positive neurons emerged at E12.5-E13.5. This same relationship was observed using single-cell RNA-sequencing of progressive stages of human embryonic spinal cord in work by Rayon et al. [34]. Specifically, Sagner et al. showed that the earliest-born express Onecut1–3, the next-born express Pou2f2 and Zfhx2–4, and the latest-born express Neurod2/6, Tcf4 and Nfia/b/x [46]. These studies suggested that temporal transcription factor codes can be used as proxies of neuronal birthdate, facilitating the classification of neurons of each progenitor domain into three common overarching epochs.

Birthdate and its associated molecular signatures then predict fundamental aspects of spinal cord structure. Osseward et al. found that the earlier-born Zfhx þ neurons occupy more ventral and lateral portions of the spinal cord while Nfi þ neurons occupy more medial portions of the spinal cord, with little overlap between them. More dramatically, they showed that long-distance projecting neurons express Zfhx genes while those expressing Neurod- and Nfi-genes generally project locally [29] (Figure 1). This phenomenon has been observed in restricted spinal lineages as well. Hayashi et al., through retrograde tracing using rabies virus, showed that earlier-born lateral v2a neurons were more likely to project to the brainstem than later-born medial v2a neurons [22]. Roome et al. found that anterolateral tract projection neurons were almost exclusively earlier-born Phox2a þ dI5 neurons [47]. In the absence of the early temporal transcription factor Pou2f2, these neurons no longer assume their position in the superficial dorsal horn [48]. Deska-Gauthier et al. showed that dorsal clusters of v3 neurons, which project rostrally in the cord, are born comparatively early amongst the general v3 population [49]. Moreover, the V1 and dI6 cardinal classes have also been shown to include neuron subtypes of different birthdates [25,50].

It is possible that birthdate plays a more general role in predicting spinal circuit structure, such that early born neurons are more likely to be last-order neurons — either projecting to other regions or serving as the penultimate output neurons of motor circuits. For example, amongst V1 class neurons, early born Renshaw cells are more likely to be premotor compared with later born local Sp8expressing cells [21], lateral v2a neurons are more likely to be premotor than medial v2a neurons [22], and early born v3 neurons are more likely to be premotor neurons than the later born (and medially located) subset [51].

## Conclusion

### Place and time together

We have considered the role of place (or dorso-ventral location) and time (or neuronal birthdate) in separate sections above, but actually these are tightly linked in vertebrate evolution and development [52]. From cartilaginous fish, through bony fish, then from amphibians and reptiles to mammals, the ventral horn is relatively conserved structurally while there is a massive and progressive expansion of the dorsal horn [53]. The dorsal horn is also formed later in embryogenesis [54]. Early neurons born in either the dorsal or ventral progenitor domains settle in the ventral horn, as well as the lateral deep dorsal horn. Then a later phase of neurogenesis gives rise to the dorsal horn, including lamina I-IV and medial lamina V-VI, with comparatively minor late-born additions to the ventral horn [31,55]. These combined processes may have allowed for separate forces to operate on cell types in the dorsal horn versus ventral horns. This could have created a hierarchical ontogeny in which the dorsal horn is organized into molecularly distinct types, while the ventral horn can be divided by birthdate and lineage into numerous populations that then converge transcriptionally after birth.

### Outstanding questions

Experiments using single cell sequencing will continue to uncover important aspects of neuronal diversity in the spinal cord. Comparative studies covering a broader range of lifespan timepoints and species will reveal the ontological and evolutionary principles at work. The addition of single cell epigenetics will detail candidate transcriptional networks that drive cell type diversity; even specific cell type features such as long-range growth or particular plasticity mechanisms may become clear. Target areas for study include probing the relationships between cardinal classes and adult cell types in the ventral horn and delineating the developmental logic of later born cell types in the dorsal horn. Both of these questions may benefit from a cell “tree” perspective recently proposed by Domcke and Shendure [16]. The influence of extrinsic factors on cell type diversity should also be explored, including from sensory pathways and descending systems, animal behavior and circuit activity, and a wide range of pathological processes from disease to injury.

The explosion of single cell sequencing data has also prompted a reconsideration of what a “cell type” even means, and studies on the spinal cord are well poised to provide important insights. Are there truly coherent categories of cells that are each distinguished by the unique set of features that they share, such as location, birthdate, lineage, gene expression, connectivity, and physiology? The difference in organization between the dorsal and ventral horns suggests that there is a spectrum of whether cells can (or should) be classified into types or considered as part of looser structures or heterogeneous populations. Emerging techniques that blend single cell sequencing with characterization of other cell types features will help to address this question, including the combination of cell type identity with either circuit tracing [56] or physiology and neural activity [57–59].

And what is the point of all this neural diversity? The prevailing model has been that each cell type has a particular function, and this may very well be true. Indeed, many studies have perturbed particular populations of spinal neurons and characterized the behavioral phenotypes that result. But to truly test this hypothesis, it is important to probe transcriptional cell types and not only the set of cells marked by a single gene. This may require a new generation of intersectional genetic and viral tools, guided by knowledge gleaned from single cell sequencing.

Finally, we return to our initial question and consider “what is the spinal cord made of?” Our review has focused on the transcriptomic classification of mammalian neuron types, but we close by placing this analysis in a wider perspective. We suggest that the spinal cord is built up from a small set of archetypal cell classes whose essential features are their neurotransmitter status and their connectivity. In this context, gene expression modules serve mainly to establish and maintain these patterns, such that a collection of genes may confer glutamatergic status, the axon guidance machinery to cross the midline, or the calcium buffering capacity to withstand high frequency sensory drive. Sequential waves of neurogenesis then expand upon these core types, leading to cellular redundancy, the grist for evolution’s mill. With this diversification, the spinal cord is then made of both old and new cell types, maintaining robust functions and allowing for novel adaptation.

## Data Availability

No data was used for the research described in the article.
